# Anatomic reconstruction of lateral ankle ligaments: is there an optimal graft option?

**DOI:** 10.1007/s00167-022-07071-7

**Published:** 2022-08-02

**Authors:** Pietro Spennacchio, Romain Seil, Caroline Mouton, Sebastian Scheidt, Davide Cucchi

**Affiliations:** 1 Department of Orthopaedic Surgery, Centre Hospitalier de Luxembourg-Clinique d’Eich, Luxembourg, Luxembourg; 2 Sports Medicine Research Laboratory, Luxembourg Institute of Health, Luxembourg, Luxembourg; 3 Department of Orthopaedics and Trauma Surgery, Universitätsklinikum Bonn, Bonn, Germany

**Keywords:** Chronic ankle instability, Ankle ligament reconstruction, Autograft, Allograft, Validated outcome, Satisfaction, Karlsson score

## Abstract

**Purpose:**

Different graft options are available for the reconstruction of lateral ankle ligaments to treat chronic ankle instability (CAI), which fall in two categories: allografts and autografts. This study aims to provide an updated comparison of the clinical outcomes after stabilisation procedures using allografts and autografts, to correctly advise the clinician during the choice of the best material to be used for the reconstruction of the lateral ligamentous complex of the ankle.

**Methods:**

A systematic review was performed to analyse the use of autografts and allografts for anatomic reconstruction of the lateral ligamentous complex of the ankle in CAI patients. The presence of a postoperative assessment through outcome measures with proofs of validation in the CAI population or patient’s subjective evaluation on the treatment were necessary for inclusion. The quality of the included studies was assessed through the modified Coleman Methodology Score (mCMS). Relevant clinical outcome data were pooled to provide a synthetic description of the results in different groups or after different procedures.

**Results:**

Twenty-nine studies (autograft: 19; allograft: 9; both procedures: 1) accounting for 930 procedures (autograft: 616; allograft: 314) were included. The average mCMS was 55.9 ± 10.5 points. The Karlsson-Peterson scale was the most frequently reported outcome scale, showing a cumulative average post- to preoperative difference of 31.9 points in the autograft group (*n* = 379, 33.8 months follow-up) and of 35.7 points in the allograft group (*n* = 227, 25.8 months follow-up). Patient satisfaction was good or excellent in 92.8% of autograft (*n* = 333, 65.2 months follow-up) and in 92.3% of allograft procedures (*n* = 153, 25.0 months follow-up). Return to activity after surgery and recurrence of instability were variably reported across the studies with no clear differences between allograft and autograft highlighted by these outcomes.

**Conclusions:**

The systematic analysis of validated CAI outcome measures and the patient’s subjective satisfaction does not support a specific choice between autograft and allograft for the reconstruction of the ankle lateral ligamentous complex in CAI patients. Both types of grafts were associated to a postoperative Karlsson–Peterson score superior to 80 points and to a similar rate of patient’s subjective satisfaction.

**Level of evidence:**

Level IV.

## Introduction

Lateral ankle sprain (LAS) is the most common acute injury of the musculoskeletal system, with high incidences especially among physically active individuals [24]. The observed high reinjury rate after a LAS is associated with a progressive insufficiency of the lateral ankle ligament complex, able to lead to the development of functional chronic ankle instability (CAI) with variable incidences that, depending on the diagnostic definition and the analysed population, can be as high as 70% [41, 64].

When comprehensive nonsurgical measures fail in patients with symptoms of instability associated to mechanical ligamentous insufficiency, surgery should be considered in order to restore a proper function of the ankle joint. The direct repair of the anterior talofibular ligament (ATFL) and the calcaneofibular ligament (CFL) with the possible addition of the inferior extensor retinaculum, referred to as the Broström–Gould procedure, represents the treatment of choice to treat CAI [6, 23, 36].

**Fig. 1:**
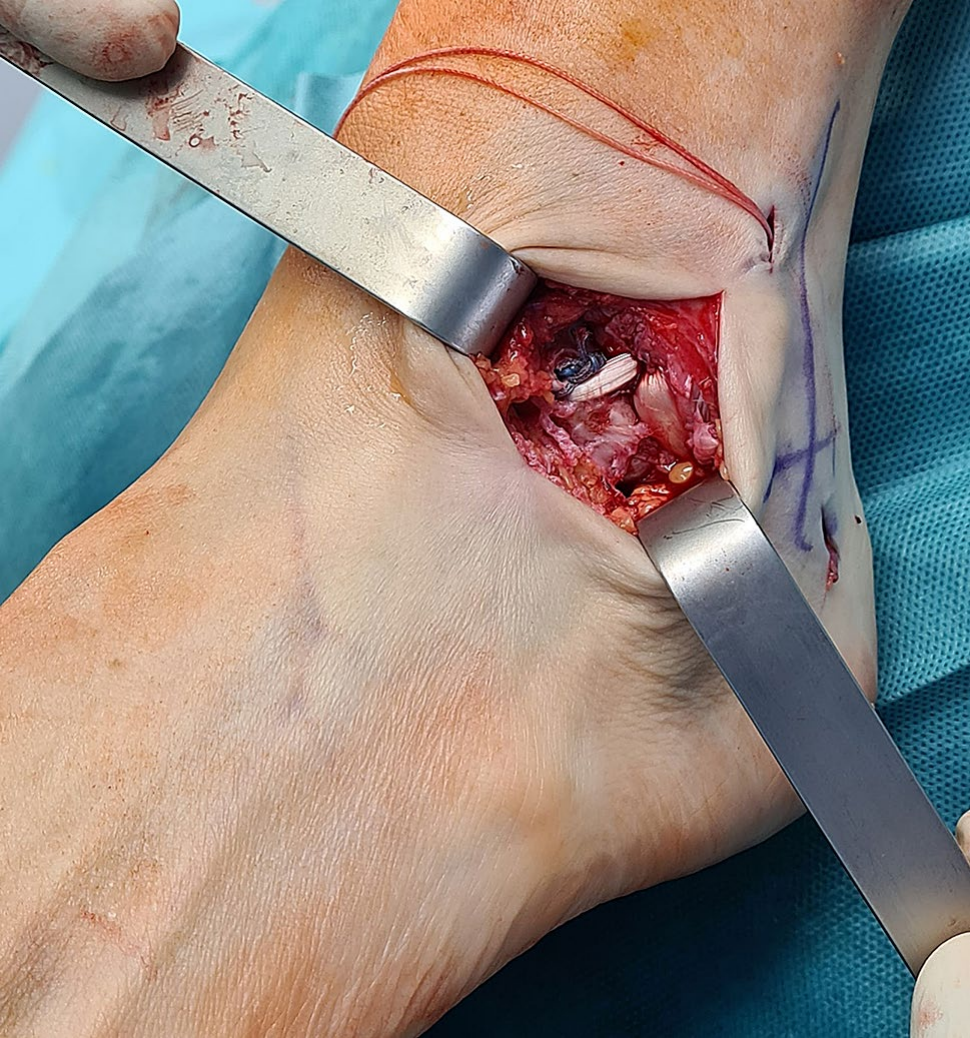
Intraoperative picture of combined ATFL/CFL anatomic reconstruction with autologous semitendinosus graft

However, some patient-related factors such as long-standing instability with degenerated lateral ligaments, failed primary stabilization procedures, and generalized ligamentous laxity, are associated with less satisfactory results after the Broström procedure [36, 51, 60]. When the ligament remnants are judged as inadequate to achieve a substantial repair of the lateral ligamentous complex, the anatomic reconstruction using a free tendon graft is considered an appropriate choice, as witnessed by clinical and biomechanical observation [47, 64] (Fig. 1).

Different grafts are available for the reconstruction of lateral ankle ligaments, which can be split into two main categories, allograft and autograft, each with advocated advantages and disadvantages but no recognized superiority of either of the two options. Currently the clinical evidence to support the selection of the optimal graft in ankle stabilization procedures is scarce and often collected through modalities not validated in the specific CAI population, which raises concern about the clinical usefulness of part of the available literature [20, 59]. This suggests that the identification of those data collected through modalities relevant for the specific CAI population could improve an evidence-based approach to different aspects of CAI surgery, such as the choice of the graft for the reconstruction of the lateral ankle ligaments.

This study aims to systematically compare the use of allograft and autograft in ankle ligament surgery, with a specific focus on the outcomes relevant to the unique CAI population. The purpose is to present an updated insight in order to make the best choice when selecting the graft to be used to reconstruct lateral ankle ligaments.

## Materials and methods

The preferred reporting items for systematic reviews and meta-analysis statement (PRISMA) was followed as a guideline for the study [44]. This study did not require ethic committee approval.

### Search strategy

To analyse the use of autografts and allografts for anatomic reconstruction of the lateral ligamentous complex of the ankle in CAI patients, a systematic review of the PubMed/Ovid Medline electronic database was performed. The specific search terms and how they were combined are reported in the Appendix. The search string was constructed with the aid of an experienced librarian with expertise in electronic searches at the Sahlgrenska University Hospital Library (University of Gothenburg, Sweden).

The search was performed on the 15th of June 2021. Combining the search terms, with no time limit and language selection (only English publications) resulted in 1365 articles.

Two reviewers (PS and DC) independently applied the below mentioned predefined inclusion and exclusion criteria to all 1365 articles.

The references of all the fully assessed articles and relevant review papers were also hand searched for the identification of additional articles.

### Eligibility criteria and study selection

#### Inclusion criteria

1. Interventional studies investigating the outcomes of the anatomical reconstruction of the lateral ligamentous complex (ATFL ± CFL) of the ankle with a free tendon graft, either allograft or autograft, either open or arthroscopic
2. Presence of a clear description of the technique (including the selected graft).
3. Postoperative outcome assessed through modalities relevant to the CAI population with a minimum 1-year follow-up.

Studies describing combined CAI and associated lesion treatment (OCD, impingement, peroneal tendinopathy) were considered for inclusion provided that CAI was the primary diagnosis and the type of procedure and the postoperative evaluation were as described in the inclusion criteria 1 and 2.

#### Exclusion criteria

1. Studies reporting on both autograft and allograft procedures without clear distinction of the outcomes obtained for each graft.
2. Studies reporting on surgical outcome after non-anatomical procedures (e.g., tenodesis).
3. Reviews, systematic reviews and meta-analyses, animal studies and ex-vivo studies.

The definition of relevant evaluation modalities necessary for inclusion entailed as minimum requirement one of the following:

- a postoperative assessment through outcome measures with proofs of validation in the CAI population;
- the patient’s subjective evaluation on the treatment (PSS), expressed in terms of subjective overall satisfaction about the results of the surgical procedure.

Available knowledge on the clinimetric qualities of patient’s related outcome measures (PROMs) in the CAI population [20], the evidence-based position statements for patient’s selection in CAI research [24] and a recent systematic review on the evaluation modalities reported in the published literature on CAI treatment [59] were used to create a list of outcome tools deemed as relevant in the CAI population: the AJFAT [56], the CAIS [19], the CAIT [28], the FAAM [11], the FAOS [55], the FADI [27], the IdFAI [58] and the Karlsson–Peterson Score [37]. Every time a score different than the aforementioned was encountered two authors independently verified the existence of proofs of validation of such instruments in the specific CAI population before the final decision was made.

#### Collected outcomes

The mentioned relevant evaluation modalities were the primary outcome of the review.

The return to activity after the surgery, the recurrence rate of instability and surgery related complications were the secondary outcomes.

#### Quality assessment

All the studies that met the final inclusion criteria were individually assessed for their quality by two independent reviewers (PS, DC) using the modified Coleman Methodology Score (mCMS). This instrument was first published in 2000 to evaluate methodological quality in studies reporting surgical outcome after patellar tendinopathy. Two modified version of the CMS were subsequently proposed to better analyse the quality of studies on cartilage repair around the knee and on osteochondral lesions of the talus [33, 54]. The latter version, which was already used also to evaluate methodologic quality of clinical outcome studies on cartilage repair of the ankle and arthroscopic repair of lateral ankle ligament for chronic lateral ankle instability, was deemed appropriate for quality assessment in this systematic review [7, 53]. Methodological quality was graded as excellent (above 85 points), good (70 to 84 points) fair (55 to 69 points), and poor (below 55 points) [33, 54].

#### Data extraction, grouping and analysed variables

Information regarding authors, journal and year of publication, study design and quality of evidence, patient demographics, indication for surgery, surgical technique, type of graft used, follow-up duration, outcome assessment instruments used and numerical outcomes, return to activity after surgery, recurrence of instability and other complications were extracted and entered into a spreadsheet for analysis.

All the included articles were then categorised depending on the type of graft used during the surgical procedure. If results for both autograft and allograft procedures were separately reported within a study, these were extracted and separately analysed in the appropriate group.

The postoperative value and the post- to preoperative change in the retained outcome measure, the return to activity and the adverse events were extracted and analysed. For the patients’ subjective satisfaction, a dichotomous distinction between the groups of “Excellent/very satisfied” + “Good/satisfied” and “Fair” + “Poor/dissatisfied” was performed to allow for comparison between studies.

### Statistical analysis

Statistical analysis was performed using GraphPad Prism v 6.0 software (GraphPad Software Inc.) and Microsoft Excel (Microsoft Corporation). The Shapiro–Wilk normality test was used to evaluate the normal distribution of the sample. Continuous variables were expressed as median and interquartile range [first and third quartiles] or mean ± standard deviation as appropriate. Dichotomous variables were expressed in numbers of cases and frequencies.

Relevant clinical outcome data from studies reporting outcome of autograft and allograft procedures in different studies were pooled to provide a synthetic description of the results in different groups or after different procedures.

## Results

### Review process and included studies

**Fig. 2:**
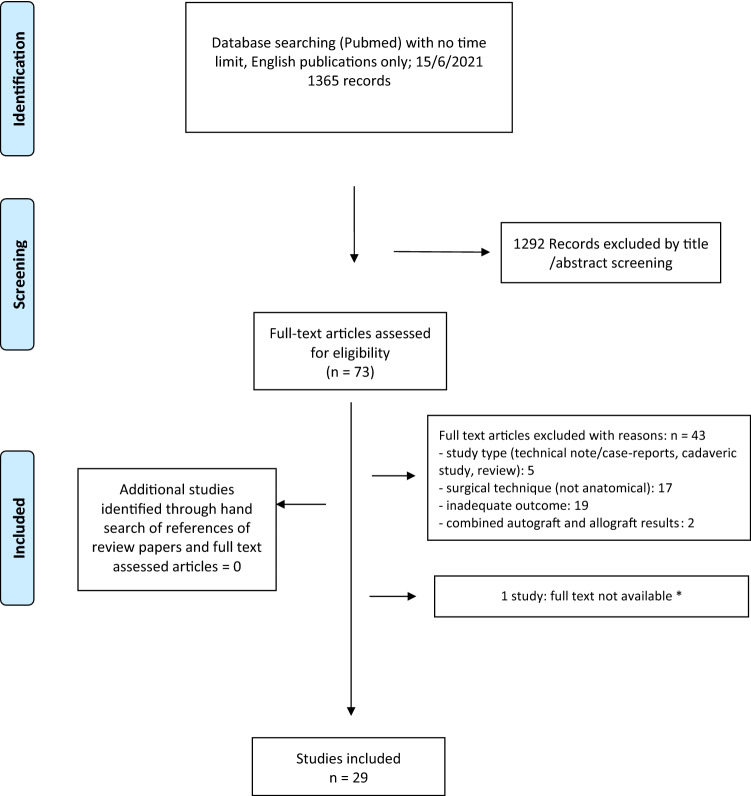
Flow chart: selection of publications for the systematic review; *Cass JR (1985) Ankle instability: comparison of primary repair and delayed reconstruction after long-term follow-up study. Clin Orthop Relat Res 198:110–117

The database search identified 1365 studies. After title and abstract screening, 73 articles were selected. The full text assessment with additional hand search of references identified 29 studies accounting for 930 procedures that were then included in this review (Fig. 2).

### Graft types

Nineteen studies reported on autograft procedures [1, 8, 9, 12, 15, 16, 22, 25, 32, 38, 39, 48–50, 52, 61, 63, 66, 67], nine on allograft procedures [10, 17, 21, 29, 34, 35, 42, 45, 65] and one on both procedures, providing separate outcome description for patients receiving autograft and allograft [43]. Overall, 616 patients received autograft reconstruction and 314 allograft reconstruction.

The distribution of selected free tendon grafts over the 930 included patients is reported in Table 1. The data describe a clear higher variation in the type of selected autograft, whereas in the allograft group 96% of the reconstruction have been realized with the semitendinosus. The semitendinosus tendon was the only free tendon graft used both as an autograft and allograft.

**Table 1:** Distribution of the graft type among the 29 included studies

	Autograft			Allograft		
	ATFL N.Pt. (N.St.)	ATFL + CFL N.Pt. (N.St.)	Sum	ATFL	ATFL + CFL	Sum
Semitendinosus	84 (*3* [22, 43, 51])	46 (3 [43, 61, 66])	130	11 (1 [43])	292 (9*)	303
Gracilis	33 (*1* [47])	143 (5**)	176	–	–	0
Plantaris	67 (*2* [8, 63])	22 (1 [8])	89	–	–	0
Palmaris longus	59 (*2* [48, 67])	–	59	–	–	0
4th toe extensor	3 (*1* [1])	20 (1 [1])	23	–	–	0
Free PL	57 (*1* [38])	59 (2 [39, 49])	116	–	–	0
TT–PT	23 (*1* [12])	–	23	–	–	0
Tibialis anterior	–	–	0	–	11 (1 [21])	11
All tendons	326	290	616	11	303	314

The number of included patients (N.Pt.) is reported first. The number of included studies (N.St.) is reported in brackets and in italic

*ATFL* anterior talofibular ligament, *CFL* calcaneofibular ligament, *PL* peroneus longus, *TT-PT* tibial tuberosity–patellar tendon

References: *[10, 17, 29, 34, 35, 42, 43, 45, 65]; **[9, 15, 16, 25, 32]

### Quality of Evidence appraisal

The average mCMS for all included studies was 55.9 ± 10.5 points (range: 33–83 points), with a slightly higher score for the autograft studies (average mCMS: 58.4 ± 10.4) as compared to the allograft studies (average mCMS: 53.1 ± 7.2). A wide variability in the methodological quality of the studies was encountered, with most of the studies ranking in “poor” or “fair” categories. Reasons for low scores were mainly the retrospective study designs, the lack of clearly reported diagnostic and inclusion criteria, as well as the low number of included patients and the short follow-up.

### Patient’s related outcomes

The distribution of the relevant CAI outcomes among the included studies is detailed in Table 2. The Karlsson-Peterson scale was the most frequently reported, used in 458 patients (16 studies) and 238 patients (7 studies) undergone to autograft and allograft procedures, respectively.

**Table 2:** Distribution of relevant CAI outcomes among the included studies

	Karlsson–Peterson score		FAOS		FADI		PSS	mCMS
	Pre- and post-op	Post-op only	Pre- and post-op	Post-op only	Pre- and post-op	Post-op only		
Autograft	13*	3 [16, 32, 63]	1 [38]	–	–	–	11 ^§^	58.42 ± 10.37
Allograft	6**	1 [21]	–	1 [21]	–	1 [45]	6 ^§§^	53.11 ± 7.22

*CAI* chronic ankle instability, *FAOS* foot and ankle outcome score, *FADI* foot and ankle disability index, *PSS* patient’s subjective evaluation on the treatment, *mCMS* modified Coleman Methodology Score

References: *[1, 9, 12, 15, 22, 39, 43, 47–49, 61, 66, 67]; **[29, 34, 35, 42, 43, 65]; ^§^[8, 9, 12, 15, 16, 25, 32, 39, 51, 61, 63]; ^§§^[10, 17, 21, 34, 35, 45]

Postoperative, an average cumulative Karlsson-Peterson score of 91.5 points in the autograft and of 88.7 in the allograft group was documented. In all but three studies in the autograft [16, 32, 63] and one in the allograft group [21], both pre- and postoperative Karlsson-Peterson score were reported, allowing for cumulative evaluation of the post-operative improvement. The pre-operative Karlsson-Peterson score was 59.5 in the autograft and 53.4 in the allograft group, with cumulative average post- to preoperative difference of 31.9 points in the autograft group (379 patients, with a cumulative average follow-up time of 33.82 months [1, 9, 12, 15, 22, 39, 43, 48–50, 61, 66, 67]) and of 35.7 points in the allograft group (227 patients, with a cumulative average follow-up time of 25.82 months [29, 34, 35, 42, 43, 65]).

The patient’s subjective satisfaction about the results of the surgical procedure was reported in 486 patients across 11 [8, 9, 12, 15, 16, 25, 32, 39, 52, 61, 63] and 6 [10, 17, 21, 34, 35, 45] studies dealing with autograft and allograft procedures, respectively. Sixteen out of 17 studies reported it on a four-point Likert scale with the categories “Excellent/very satisfied”, “Good/satisfied”, “Fair” and “Poor/dissatisfied”, enabling further data analysis: cumulative results for subjective satisfaction showed good or excellent results in 92.8% of 333 patients undergoing autograft reconstruction with a cumulative average follow-up time of 65.2 months and in 92.3% of 153 patients undergoing allograft reconstruction with a cumulative average follow-up time of 25.0 months. One study [45] reported satisfaction on a modified Likert scale of 1 to 10, reporting results as median and range and was not grouped in the aforementioned categories.

Other retained valid CAI scores were the FAOS and the FADI used in two and one study, respectively, with no possible cumulative data analysis [21, 38, 45].

A detailed overview of the number of patients evaluated with each outcome measure, grouped for different graft types, is presented in Table 3.

**Table 3:** Cumulative relevant CAI outcome scores grouped for different graft type

	Semitendinosus				Gracilis		Plantaris	
	Autograft N.Pt. (N.St.)	Postop. (Δ)/%^a^	Autograft N.Pt. (N.St.)	Postop. (Δ)/%^a^	Autograft N.Pt. (N.St.)	Postop. (Δ)/%^a^	Autograft N.Pt. (N.St.)	Postop. (Δ)/%^a^
Karlsson	81 (*3* [22, 43, 66])	89.6 (31.9)	227 (*6*^b^)	89.0 (35.7)	176 (*6*^c^)	92.7 (32.8^d^)	37 (*1* [63])	85 (n.a.^e^)
FAOS	–	–	–	–	–	–	–	–
FADI	–	–	21 (*1* [45])	91 (n.a.^e^)	143 (*5* [9, 15, 16, 25, 32])	–	–	–
PSS	49 (*2* [51, 61])	91.8%	144 (*4* [10, 17, 34, 35])	92.4%	143 (*5* [9, 15, 16, 25, 32])	95.1%	89 (*2* [8, 63])	91.0%

	Palmaris longus		Extensor of the 4th toe		Free PL		TT–PT		Tibialis anterior	
	Autograft N.Pt. (N.St.)	Postop. (Δ)/%^a^	Autograft N.Pt. (N.St.)	Postop. (Δ)/%^a^	Autograft N.Pt. (N.St.)	Postop. (Δ)/%^a^	Autograft N.Pt. (N.St.)	Postop. (Δ)/%^a^	Autograft N.Pt. (N.St.)	Postop. (Δ)/%^a^
Karlsson	59 (*2* [48, 67])	97.4 (29.2)	23 (*1* [1])	92.2 (44.2)	59 (*2* [39, 49])	88.7 (26.0)	23 (*1* [12])	91.2 (36.0)	11 (*1* [29])	82.3 (n.a.^e^)
FAOS	–	–	–	–	57 (*1* [38])	91.0 (n.a.^e^)	–	–	11 (*1* [29])	78.6 (n.a.^e^)
FADI	–	–	–	–	–	–	–	–	–	–
PSS	–	–	–	–	29 (*1* [39])	83.8%	–	–	11 (*1* [29])	90.9%

*CAI* chronic ankle instability, *FAOS* foot and ankle outcome score, *FADI* foot and ankle disability index, *PSS* patient’s subjective evaluation on the treatment, *PL* palmaris longus, *TT–PT* tibial tuberosity–patellar tendon

^a^This column indicates the cumulative postoperative value for the reported scale: Karlsson–Peterson, FAOS, FADI and, in brackets, the average post- to preoperative difference for the studies furnishing the preoperative data. The percentage in the fourth line of the table refers to the patient’s rate of good to excellent subjective satisfaction

^b^[29, 34, 35, 42, 43, 65]

^c^[9, 15, 16, 25, 32, 47]

^d^Two studies (42 Patients) were excluded for lack of preoperative Karlsson-Peterson score-[16, 32]

^e^Only postoperative values were reported, thus not allowing to calculate a post- to preoperative difference [29, 38, 45, 63]

### Return to activity

The functional activity level after surgery was reported in fifteen out of the 29 included studies (52%) through different criteria. Nine studies on autograft procedures analysed the return to preinjury activity level over 296 patients with reported rate between 76 and 100% [9, 15, 25, 38, 49, 50, 52, 61, 67], with 4 studies (116 patients) providing description of the preinjury activity level [25, 49, 61, 67]. Only two studies analysed the physical activity after allograft ligament reconstruction [17, 45], one of which reporting 58% return to preinjury activity level after combined ATFL/CFL reconstruction with semitendinosus in 31 patients, with preoperative evaluation of the activity level through the Tegner scale [17].

### Instability recurrence

Twelve out of the 29 included studies (41%) did not mention the recurrence of subjective instability among the postoperative evaluations. Thirteen studies on autograft procedures analysed this specific outcome [8, 9, 12, 15, 16, 25, 39, 43, 49, 52, 63, 66, 67]: 8 studies described 0% of recurrency over 222 patients [12, 15, 16, 25, 42, 49, 66, 67], whereas the remaining 5 studies (161 patients) reported within-study recurrency rates ranging from 2 to 30% [8, 9, 39, 52, 63].

Residual subjective instability was analysed in 105 patients over 5 studies dealing with allograft procedures. Three studies reported 0% recurrency rate over a total of 70 patients [17, 34, 43]. The remaining two studies observed a recurrency rate of 8% (24 patients) and 18% (11 patients) after combined ATFL/CFL reconstruction with allogenic semitendinosus and anterior tibialis tendon graft, respectively [10, 21].

### Complications

Twenty-four studies analysed the occurrence of surgery-related complications over 527 patients undergoing autograft procedures (18 studies [1, 9, 12, 15, 16, 22, 25, 32, 38, 39, 43, 48–50, 52, 61, 66, 67]) and 249 patients undergoing allograft procedures (7 studies [10, 17, 34, 35, 42, 43, 45]).

Postoperative infections were reported in 5/527 patients after autograft procedures with within-study incidences from 4.3% to 8.7% [16, 43, 61] and in 5/249 patients over three studies on allograft procedures with within-study  incidences from 1.5% to 4.2% [17, 35, 42].

Hardware related problems were noted in 12/527 autograft procedures, namely tenderness at the insertion of bioabsorbable screws in six patients [39, 50], and six cases of discomfort/irritation due to the suspension device used for fibular fixation of the graft [15, 25].

In the allograft group, two patients enrolled in the same study reported soft tissue irritation related to the fibular suspension device [10].

Fifteen out of the 527 patients (2.9%) undergoing allograft procedures suffered from harvesting-related problems: 9 cases of peri-incisional paraesthesia around the proximal tibia after hamstring or patellar tendon harvesting [12, 16, 52], 3 cases of dorsiflexion toe weakness after extensor of the 4th toe harvesting [1], 1 case of peroneus longus tendinopathy after half peroneus longus harvesting [38], 1 case of knee superficial wound infection [9] and 1 case of unspecified knee pain both at the hamstring harvest site [61].

No serious adverse events related to allograft use, such as reject or immune reaction to the allograft, were reported.

## Discussion

The most important finding of this study is that the systematic analysis of validated CAI outcome measures and the patient’s subjective satisfaction does not support a specific choice between autograft and allograft for the reconstruction of the ankle lateral ligamentous complex in CAI patients.

Both types of grafts were always associated in the included literature to a postoperative Karlsson-Peterson score superior to 80 points, which is the value described in the original score validation study as the threshold for good/excellent results [37]. Besides, the patient’s subjective report about the results of surgery confirmed similarly high rates of satisfaction (> 90%) associated with both autograft and allograft.

The retrieved data are in accordance with the available knowledge on the use of allografts and autografts for ankle ligaments surgery [5, 62, 64]. Brambilla et al. also concluded that the available peer-reviewed literature does not ascertain superiority of either of the two options [5]. However, the cumulative analysis of the validated CAI outcome measures selected for this review to compare the use of allograft and autograft had not been performed yet and furnishes an original information to the surgeon dealing with ankle stabilisation procedures.

Besides validated outcome scales and patients’ satisfaction, three other evaluation modalities have been analysed in this review to compare allograft and autograft options: return to activity after surgery, recurrence rate and complications.

The return to physical activity after surgery is a meaningful parameter in the typically active CAI population, especially in light of the observed discrepancy between preinjury and postoperative activity level, in spite of high scores on standardized outcome scales [40, 47]. It is recognised that highly active patients are at risk for a “ceiling effect” when assessed through patient-reported outcome measures, which may poorly characterize their true recovery [13]. The patient’s subjective satisfaction with the procedure may also inadequately reflect the level of postoperative activity: May et al. [46] noticed 88% of high satisfaction after surgical stabilisation of the ankle after a Broström procedure among 41 patients of which, however, only 54% resumed their preinjury activity level. Unfortunately, half of the studies included in this review did not analyse the postoperative activity level, confirming previous observations where return to activity after ankle stabilisation procedures is poorly explored in the available literature [31, 59]. Moreover, the absence of information about physical activity recovery in the vast majority of the studies dealing with allograft procedures hindered further comparison between allograft and autograft under this noteworthy perspective.

Regarding postoperative recurrence of CAI, both autografts and allografts proved to be effective options with a similar recurrence rate and complete resolution of instability symptoms in the vast majority of treated patients, which is in accordance with the already available knowledge on residual instability after ankle stabilisation procedures [57]. However, the ununiform definition of recurrency across the included studies (from isolated traumatic ankle sprains to persisting feeling of ankle instability) hindered a consistent comparison between autograft and allograft procedures.

Complication rates also failed to identify differences between autograft and allograft procedures with similar low incidences of postoperative infections and hardware-related problems with both techniques. Furthermore, the retrieved data does not sustain the feared risk of infection transmission and immunogenic reaction related to allograft application, which is in line with previous observations on the topic [18].

Due to the seemingly absent clinical superiority of any graft for lateral ligament reconstruction, it appears reasonable to analyse basic science studies to find support for treatment selection.

From a pure biomechanical perspective, an ideal graft should match the mechanical properties of the native ligaments and be able to overcome the loss of strength occurring during the ligamentization process of the graft [26]. Available knowledge on the biomechanical features of the ankle ligaments and reconstructive tendon grafts indicate that the semitendinosus, the posterior and anterior tibialis tendons and the peroneal tendons meet or exceed the ultimate tensile strength of the native ankle ligaments [3, 4, 14]. In particular, the ex vivo anatomic reconstruction of the ATFL using a semitendinosus allograft fixed with absorbable screws demonstrated similar strength and stiffness to the intact ATFL [14]. The analyzed literature highlighted that allograft procedures are almost always performed with semitendinosus graft, which is in accordance with the mentioned biomechanical evidences. The choice of tissue source is instead more varied for autograft reconstruction with some selected tendons such as the palmaris longus, the plantaris and the 4^th^ extensor, which are not fully supported by the current biomechanical knowledge [2]. The fact that the available outcomes did not show clinical differences or inferiority of any selected graft could be attributed either to the not uncommon discrepancy between biomechanical data and clinical outcomes, the inadequacy of the current evaluation modalities to depict differences between the different techniques or the insufficient quality of the included studies.

Some theoretical specific disadvantages associated with allografts should also be considered, such as increased time to incorporation and variability in mechanical strength due to sterilization techniques and use of irradiation [26]. Knowledge derived from the broader use of grafts in knee surgery highlighted a higher risk of failure with allografts in association with irradiation for sterilization [30]. Therefore, the use of a fresh frozen and non-irradiated allograft for the reconstruction of the lateral ligamentous complex of the ankle is recommended to avoid iatrogenic weakening of the graft.

The main limitation of this systematic review is related to the quality of the included study as described by the mCMS score. The retrospective nature of the majority of the included studies (27 out of 29), the lack of clearly reported diagnostic and inclusion criteria and the heterogeneity of the selected grafts affected the consistency of data pooling and the proposed comparison between allograft and autograft procedures. The absence of preoperative scores in some studies lowered the number of patients available for pooled post- to preoperative changes assessment. This, in association with the frequently small number of patients within the studies, reduced the possibility of statistical sound comparisons between allograft and autograft groups. Likewise, it implies bias due to unknown preoperative scores when analyzing the global postoperative scores of the two groups.

Another potential limitation is related to the eligibility criteria, namely the use of predefined CAI evaluation modalities as minimum requirement for inclusion, which may have excluded additional data on secondary outcomes for comparison between allografts and autografts. Nevertheless, the 776 patients analyzed for postoperative complications furnished a broad overview of this outcome in CAI reconstruction surgery; the low morbidity observed after hamstring harvesting and the similar infection rates between autografts and allografts are concordant with a vast body of knowledge on the use of grafts in knee ligament reconstruction surgery [30]. Additional information on ankle instability recurrency might have also been retrieved from the excluded literature. Yet, previous observation on the absence of this evaluation in half of the research dealing with CAI surgery [58] and a not standardized definition of postoperative recurrency limit the concerns about the loss of this specific information in the excluded studies. In light of these observations and considering the aforementioned inherent methodological weaknesses of the literature dealing with CAI surgery, we do not expect that the studies excluded because of inadequate primary outcomes would have furnished considerably different conclusions on the comparison between allografts and autografts procedures.

The effectiveness of ankle ligament reconstruction for CAI treatment shown in this review is primary supported by a Karlsson-Peterson score which was always beyond 80 points, regardless the selected graft. This value has been suggested by the score developers to correspond to good-to-excellent postoperative results [37]. Despite specific validation of the Karlsson-Peterson score in the CAI population, the correlation of the scale values with the patient’s symptomatic state, as well as the correlation between post- to preoperative changes and the clinical benefit perceived by the patient are unknown. Future definition of significant outcomes for validated CAI scores would allow a better interpretation of the reported results eventually leading to a more reliable comparison between different strategies such as the use of allograft or autograft for reconstruction of lateral ankle ligaments.

## Conclusions

The data reported in the study support the use of both allografts and autografts for the reconstruction of lateral ankle ligaments, with no evident clinical difference highlighted by the evaluation modalities relevant to the specific CAI population. It can thus be concluded that in case of ankle stabilization procedures with a deficient native ligamentous complex both allografts and autografts can be offered to the patient, with similar expected clinical results.

## References

[1] Ahn JH, Choy W-S, Kim H-Y (2011). Reconstruction of the lateral ankle ligament with a long extensor tendon graft of the fourth toe. Am J Sports Med.

[2] Attarian DE, Mccrackin HJ, Devit DP, Mcelhaney JH, Garrett WE (1985). A biomechanical study of human lateral ankle ligaments and autogenous reconstructive grafts. Am J Sports Med.

[3] Attarian DE, McCrackin HJ, DeVito DP, McElhaney JH, Garrett WE (1985). Biomechanical characteristics of human ankle ligaments. Foot Ankle.

[4] Bohnsack M, Sürie B, Kirsch L, Wülker N (2002). Biomechanical properties of commonly used autogenous transplants in the surgical treatment of chronic lateral ankle instability. Foot Ankle Int.

[5] Brambilla L, Bianchi A, Malerba F, Loppini M, Martinelli N (2020). Lateral ankle ligament anatomic reconstruction for chronic ankle instability: allograft or autograft? A systematic review. Foot Ankle Surgery.

[6] Broström L (1966). Sprained ankles. VI. Surgical treatment of “chronic” ligament ruptures. Acta Chir Scand.

[7] Brown AJ, Shimozono Y, Hurley ET, Kennedy JG (2018). Arthroscopic repair of lateral ankle ligament for chronic lateral ankle instability: a systematic review. Arthroscopy.

[8] Brunner R, Gaechter A (1991). Repair of fibular ligaments: comparison of reconstructive techniques using plantaris and peroneal tendons. Foot Ankle.

[9] Caals M, Mertens P, Spaepen D, Buedts K (2020). Outcome after arthroscopically assisted percutaneous reconstruction of lateral ankle ligaments using a gracilis tendon autograft. Acta Orthop Belg.

[10] Cao Y, Xu Y, Hong Y, Xu X (2018). A new minimally invasive method for anatomic reconstruction of the lateral ankle ligaments with a Tightrope system. Arch Orthop Trauma Surg.

[11] Carcia CR, Martin RL, Drouin JM (2008). Validity of the foot and ankle ability measure in athletes with chronic ankle instability. J Athl Train.

[12] Chen C, Lu H, Hu J, Qiu X, Li X, Sun D, Qu J, Zhang T, Xu D (2018). Anatomic reconstruction of anterior talofibular ligament with tibial tuberosity–patellar tendon autograft for chronic lateral ankle instability. J Orthop Surg (Hong Kong).

[13] Chona DV, Bonano JC, Ayeni OR, Safran MR (2020). Definitions of return to sport after hip arthroscopy: are we speaking the same language and are we measuring the right outcome?. Orthop J Sports Med.

[14] Clanton TO, Viens NA, Campbell KJ, LaPrade RF, Wijdicks CA (2014). Anterior talofibular ligament ruptures, part 2: biomechanical comparison of anterior talofibular ligament reconstruction using semitendinosus allografts with the intact ligament. Am J Sports Med.

[15] Cordier G, Ovigue J, Dalmau-Pastor M, Michels F (2020). Endoscopic anatomic ligament reconstruction is a reliable option to treat chronic lateral ankle instability. Knee Surg Sports Traumatol Arthrosc.

[16] Coughlin MJ, Schenck RC, Grebing BR, Treme G (2004). Comprehensive reconstruction of the lateral ankle for chronic instability using a free gracilis graft. Foot Ankle Int.

[17] Dierckman BD, Ferkel RD (2015). Anatomic reconstruction with a semitendinosus allograft for chronic lateral ankle instability. Am J Sports Med.

[18] Diniz P, Pacheco J, Flora M, Quintero D, Stufkens S, Kerkhoffs G, Batista J, Karlsson J, Pereira H (2019). Clinical applications of allografts in foot and ankle surgery. Knee Surg Sports Traumatol Arthrosc.

[19] Eechaute C, Vaes P, Duquet W (2008). The chronic ankle instability scale: clinimetric properties of a multidimensional, patient-assessed instrument. Phys Ther Sport.

[20] Eechaute C, Vaes P, Van Aerschot L, Asman S, Duquet W (2007). The clinimetric qualities of patient-assessed instruments for measuring chronic ankle instability: a systematic review. BMC Musculoskelet Disord.

[21] Ellis SJ, Williams BR, Pavlov H, Deland J (2011). Results of anatomic lateral ankle ligament reconstruction with tendon allograft. HSS J.

[22] Feng S-M, Maffulli N, Oliva F, Wang A-G, Sun Q-Q (2020). Arthroscopic remnant-preserving anterior talofibular ligament reconstruction does not improve mid-term function in chronic ankle instability. Injury.

[23] Gould N, Seligson D, Gassman J (1980). Early and late repair of lateral ligament of the ankle. Foot Ankle.

[24] Gribble PA, Bleakley CM, Caulfield BM, Docherty CL, Fourchet F, Fong DT-P, Hertel J, Hiller CE, Kaminski TW, McKeon PO, Refshauge KM, Verhagen EA, Vicenzino BT, Wikstrom EA, Delahunt E (2016). Evidence review for the 2016 International Ankle Consortium consensus statement on the prevalence, impact and long-term consequences of lateral ankle sprains. Br J Sports Med.

[25] Guillo S, Odagiri H, van Rooij F, Bauer T, Hardy A (2021). All-inside endoscopic anatomic reconstruction leads to satisfactory functional outcomes in patients with chronic ankle instability. Knee Surg Sports Traumatol Arthrosc.

[26] Gulotta LV, Rodeo SA (2007). Biology of autograft and allograft healing in anterior cruciate ligament reconstruction. Clin Sports Med.

[27] Hale SA, Hertel J (2005). Reliability and sensitivity of the foot and ankle disability index in subjects with chronic ankle instability. J Athl Train.

[28] Hiller CE, Refshauge KM, Bundy AC, Herbert RD, Kilbreath SL (2006). The cumberland ankle instability tool: a report of validity and reliability testing. Arch Phys Med Rehabil.

[29] Hua Y, Chen S, Jin Y, Zhang B, Li Y, Li H (2012). Anatomical reconstruction of the lateral ligaments of the ankle with semitendinosus allograft. Int Orthop.

[30] Hulet C, Sonnery-Cottet B, Stevenson C, Samuelsson K, Laver L, Zdanowicz U, Stufkens S, Curado J, Verdonk P, Spalding T (2019). The use of allograft tendons in primary ACL reconstruction. Knee Surg Sports Traumatol Arthrosc.

[31] Hunt KJ, Fuld RS, Sutphin BS, Pereira H, D’Hooghe P (2017). Return to sport following lateral ankle ligament repair is under-reported: a systematic review. J ISAKOS.

[32] Ibrahim SA, Hamido F, Al Misfer AK, Ghafar SA, Awad A, Salem HKh, Alhran H, Khirait S (2011). Anatomical reconstruction of the lateral ligaments using Gracillis tendon in chronic ankle instability; a new technique. Foot Ankle Surg.

[33] Jakobsen RB (2005). An analysis of the quality of cartilage repair studies. J Bone Joint Surg Am.

[34] Jung H-G, Kim T-H, Park J-Y, Bae E-J (2012). Anatomic reconstruction of the anterior talofibular and calcaneofibular ligaments using a semitendinosus tendon allograft and interference screws. Knee Surg Sports Traumatol Arthrosc.

[35] Jung H-G, Shin M-H, Park J-T, Eom J-S, Lee D-O, Lee S-H (2015). Anatomical reconstruction of lateral ankle ligaments using free tendon allografts and biotenodesis screws. Foot Ankle Int.

[36] Karlsson J, Bergsten T, Lansinger O, Peterson L (1989). Surgical treatment of chronic lateral instability of the ankle joint: a new procedure. Am J Sports Med.

[37] Karlsson J, Peterson L (1991). Evaluation of ankle joint function: the use of a scoring scale. Foot.

[38] Kennedy JG, Smyth NA, Fansa AM, Murawski CD (2012). Anatomic lateral ligament reconstruction in the ankle: a hybrid technique in the athletic population. Am J Sports Med.

[39] Kim HN, Jeon JY, Dong Q, Noh KC, Chung KJ, Kim HK, Hwang JH, Park YW (2015). Lateral ankle ligament reconstruction using the anterior half of the peroneus longus tendon. Knee Surg Sports Traumatol Arthrosc.

[40] Krips R, van Dijk CN, Lehtonen H, Halasi T, Moyen B, Karlsson J (2002). Sports activity level after surgical treatment for chronic anterolateral ankle instability: a multicenter study. Am J Sports Med.

[41] Lam KC, Snyder Valier AR, Valovich McLeod TC (2015). Injury and treatment characteristics of sport-specific injuries sustained in interscholastic athletics: a report from the athletic training practice-based research network. Sports Health.

[42] Lee D-O, Eom J-S, Jung H-G (2018). The effect of smoking on the outcomes of lateral ankle ligament reconstruction. J Orthop Sci.

[43] Li Q, Ma K, Tao H, Hua Y, Chen S, Chen S, Zhao Y (2018). Clinical and magnetic resonance imaging assessment of anatomical lateral ankle ligament reconstruction: comparison of tendon allograft and autograft. Int Orthop.

[44] Liberati A, Altman DG, Tetzlaff J, Mulrow C, Gøtzsche PC, Ioannidis JPA, Clarke M, Devereaux PJ, Kleijnen J, Moher D (2009). The PRISMA statement for reporting systematic reviews and meta-analyses of studies that evaluate health care interventions: explanation and elaboration. PLoS Med.

[45] Matheny LM, Johnson NS, Liechti DJ, Clanton TO (2016). Activity level and function after lateral ankle ligament repair versus reconstruction. Am J Sports Med.

[46] May NR, Driscoll M, Nguyen S, Ferkel RD (2022). Analysis of return to play after modified Broström lateral ankle ligament reconstruction. Orthop J Sports Med.

[47] Michels F, Pereira H, Calder J, Matricali G, Glazebrook M, Guillo S, Karlsson J, Acevedo J, Batista J, Bauer T, Calder J, Carreira D, Choi W, Corte-real N, Glazebrook M, Ghorbani A, Giza E, Guillo S, Hunt K, Karlsson J, Kong SW, Lee JW, Michels F, Molloy A, Mangone P, Matsui K, Nery C, Ozeki S, Pearce C, Pereira H, Perera A, Pijnenburg B, Raduan F, Stone J, Takao M, Tourné Y, Vega J, Group TE-AAI (2018) Searching for consensus in the approach to patients with chronic lateral ankle instability: ask the expert. Knee Surg Sports Traumatol Arthrosc 26:2095–2102 10.1007/s00167-017-4556-0 28439639

[48] Miyamoto W, Takao M, Yamada K, Matsushita T (2014). Accelerated versus traditional rehabilitation after anterior talofibular ligament reconstruction for chronic lateral instability of the ankle in athletes. Am J Sports Med.

[49] Okuda R, Kinoshita M, Morikawa J, Yasuda T, Abe M (2005). Arthroscopic findings in chronic lateral ankle instability: do focal chondral lesions influence the results of ligament reconstruction?. Am J Sports Med.

[50] Park CH, Lee W-C (2017). Donor site morbidity after lateral ankle ligament reconstruction using the anterior half of the peroneus longus tendon autograft. Am J Sports Med.

[51] Park KH, Lee JW, Suh JW, Shin MH, Choi WJ (2016). Generalized ligamentous laxity is an independent predictor of poor outcomes after the modified broström procedure for chronic lateral ankle Instability. Am J Sports Med.

[52] Paterson R, Cohen B, Taylor D, Bourne A, Black J (2000). Reconstruction of the lateral ligaments of the ankle using semi-tendinosis graft. Foot Ankle Int.

[53] Pinski JM, Boakye LA, Murawski CD, Hannon CP, Ross KA, Kennedy JG (2016). Low level of evidence and methodologic quality of clinical outcome studies on cartilage repair of the ankle. Arthroscopy.

[54] Ramponi L, Yasui Y, Murawski CD, Ferkel RD, DiGiovanni CW, Kerkhoffs GMMJ, Calder JDF, Takao M, Vannini F, Choi WJ, Lee JW, Stone J, Kennedy JG (2017). Lesion size is a predictor of clinical outcomes after bone marrow stimulation for osteochondral lesions of the talus: a systematic review. Am J Sports Med.

[55] Roos EM, Brandsson S, Karlsson J (2001). Validation of the foot and ankle outcome score for ankle ligament reconstruction. Foot Ankle Int.

[56] Rozzi SL, Lephart SM, Sterner R, Kuligowski L (1999). Balance training for persons with functionally unstable ankles. J Orthop Sports Phys Ther.

[57] Sammarco VJ (2001). Complications of lateral ankle ligament reconstruction. Clin Orthop Relat Res.

[58] Simon J, Donahue M, Docherty C (2012). Development of the identification of functional ankle instability (IdFAI). Foot Ankle Int.

[59] Spennacchio P, Meyer C, Karlsson J, Seil R, Mouton C, Senorski EH (2020). Evaluation modalities for the anatomical repair of chronic ankle instability. Knee Surg Sports Traumatol Arthrosc.

[60] Stewart DR (2004). Does generalised ligamentous laxity increase seasonal incidence of injuries in male first division club rugby players?. Br J Sports Med.

[61] Usuelli FG, Indino C, Di Silvestri CA, Manzi L, Maffulli N (2021). Clinical outcomes and return to sport after minimally invasive reconstruction of the lateral ligament complex with semitendinosus tendon autograft in chronic lateral ankle instability. J Am Podiatr Med Assoc.

[62] Vopat ML, Lee B, Mok AC, Hassan M, Morris B, Tarakemeh A, Zackula R, Mullen S, Schroeppel P, Vopat BG (2022). Primary repair, reconstruction, and suture tape augmentation all provide excellent outcomes for lateral ligament instability: a systematic review. Arthrosc Sport Med Rehabil.

[63] Vries JSD, Struijs PAA, Raaymakers ELFB, Marti RK (2005). Long-term results of the Weber operation for chronic ankle instability: 37 patients followed for 20–30 years. Acta Orthop.

[64] Vuurberg G, Pereira H, Blankevoort L, van Dijk CN (2018). Anatomic stabilization techniques provide superior results in terms of functional outcome in patients suffering from chronic ankle instability compared to non-anatomic techniques. Knee Surg Sports Traumatol Arthrosc.

[65] Wang W, Xu GH (2017). Allograft tendon reconstruction of the anterior talofibular ligament and calcaneofibular Ligament in the treatment of chronic ankle instability. BMC Musculoskelet Disord.

[66] Wei S, Fan D, Han F, Tang M, Kong C, Xu F, Cai X (2021). Using arthroscopy combined with fluoroscopic technique for accurate location of the bone tunnel entrance in chronic ankle instability treatment. BMC Musculoskelet Disord.

[67] Yasuda T, Shima H, Mori K, Tsujinaka S, Neo M (2017). Simultaneous reconstruction of the medial and lateral collateral ligaments for chronic combined ligament injuries of the ankle. Am J Sports Med.

